# Tiled-Amplicon Whole-Genome Sequencing Method Reveals Endemic Circulation of Human Adenovirus Type 3 in Japan

**DOI:** 10.3390/v18010074

**Published:** 2026-01-05

**Authors:** Gabriel Gonzalez, Naganori Nao, Koshiro Tabata, Yukari Itakura, Shinji Saito, Kenichiro Takahashi, Masaaki Kobayashi, Nobuyoshi Kitaichi, Nobuhisa Ishiguro, Tsuguto Fujimoto, Adriana E. Kajon, Hirofumi Sawa, Nozomu Hanaoka

**Affiliations:** 1 Institute for Vaccine Research and Development (HU-IVReD), Hokkaido University, Sapporo 001-0021, Japan; 2 Japan Institute for Health Security, National Institute for Infectious Diseases, Tokyo 162-8640, Japan; takaken@niid.go.jp (K.T.); fujimo-t@niid.go.jp (T.F.); 3 Kobayashi Pediatric Clinic, Shizuoka 426-0067, Japan; 4 Department of Ophthalmology, Health Sciences University of Hokkaido Hospital, Sapporo 002-8072, Japan; 5 Department of Pediatrics, Sophia Kita-Maruyama Clinic, Sapporo 060-0004, Japan; 6 Lovelace Biomedical Research Institute, Albuquerque, NM 87108, USA; 7 One Health Research Center, Hokkaido University, Sapporo 060-0818, Japan; 8 International Collaboration Unit, International Institute for Zoonosis Control, Hokkaido University, Sapporo 001-0020, Japan; 9 Global Virus Network, Baltimore, MD 21201, USA

**Keywords:** *Mastadenovirus blackbeardi*, human adenovirus type 3, genome sequencing, acute respiratory illness

## Abstract

Human adenovirus type 3 (HAdV-3) in the species *Mastadenovirus blackbeardi* is a frequent cause of hundreds of respiratory infections in Japan, with outbreaks varying in clinical severity. Such a high frequency of cases could be due to regular migration of novel variants or persistent circulation of endemic strains. Either scenario would require different measures to ameliorate the burden on public health. We designed a new cost-effective whole-genome sequencing protocol based on tiled amplicons and nanopore sequencing to clarify the circumstances behind the frequent outbreaks. This protocol was used with clinical samples collected between 2011 and 2020 from Japanese patients with acute respiratory illness (*n* = 110), resulting in near whole-genome sequences (~99% length) for 105 samples with high read coverage (~262.6 ± 192 reads). Phylogenetic analysis indicated sustained circulation of endemic strains in Japan during the analyzed decade and their relation to other strains worldwide with publicly available genome sequences. However, a comparison with other Japanese HAdV-3 strains circulating since 2023 suggested the public health measures introduced during the COVID-19 pandemic (2020–2022) indirectly affected the prevalence of circulating HAdV-3 variants. Additionally, our approach enabled the detection and partial sequencing (~71% completion) of co-infecting strains from the species *Mastadenovirus caesari* (*n* = 4) in the examined samples. The detection of adenoviruses belonging to different species in the same sample highlights how our protocol enhances the distinction of circulating viruses. In conclusion, these results and the introduced protocol will enable timely characterization and clinical interventions to mitigate this virus.

## 1. Introduction

Human adenovirus type 3 (HAdV-3) in the species *Mastadenovirus blackbeardi* (HAdV-B) is a common cause of respiratory and ocular infections worldwide. Severe clinical cases are found mainly in infants, the elderly, and immunocompromised patients [1,2,3]. Adenovirus accounts for 5% to 10% of acute pediatric respiratory infections, while the respiratory infection rate in adults ranges between 1% and 4% [2]. Although adenoviral infections occur year-round, many adenovirus types exhibit seasonal peaks in outbreaks. A remarkable thermostable structure of the members of the *Adenoviridae* family has facilitated the survival and transmission of the different types in closed communities [4]; for instance, as a frequent cause of nosocomial infections in Japanese hospitals [5]. In Japan, the National Institute of Infectious Diseases (NIID) maintains nationwide records of infectious viruses, including adenoviral outbreaks of respiratory, enteric, and ocular infections [6].

As a member of the *Adenoviridae* family, HAdV-3 presents a double-stranded linear genome of about 35 kilobase pairs (kbp) encapsidated in non-enveloped viral particles composed mainly of three major capsid proteins: trimeric hexon proteins (*n* = 240) forming the icosahedral capsid, pentameric penton-base proteins (*n* = 12) in the vertices with protruding trimeric fiber proteins (*n* = 12), which facilitate the virus attachment and internalization into host cells [7]. Replication of the virus occurs in the cellular nucleus by the viral-encoded DNA polymerase in coordination with the pre-terminal protein (pTP) and the DNA-binding protein (DBP). Progeny viral particles are released by cell lysis mediated by multiple pathways depending on the adenovirus species and types [8]. The main antigenic differences among types are found in epitope-determinants located on the major capsid proteins; however, differences in clinical presentation have been associated with the proteins encoded in the early transcriptional regions (E): E1, E3, and E4. The products of these early regions interact directly with various host proteins to mediate transcription of viral genes, viral genome replication, and evasion of the innate immune response [9]. The most noticeable source of diversity in the genus *Mastadenovirus* has been attributed to recombination events [10,11]. Nevertheless, accumulation of point mutations has been tracked over the years through different genomic variants recognized by restriction fragment length polymorphisms (RFLP) [12]. It is noteworthy that although this method offers advantages, such as low cost and replicability, it relies on a limited number of sites in the genome and, compared to genome sequencing, provides lower resolution into the evolutionary processes driving viral divergence.

Considering the annual occurrence of respiratory infections caused by HAdV-3 in Japan, we examined its dynamic evolution over 10 years (2011 to 2020). This analysis was intended to explore factors allowing the recurrent circulation of the virus. To this end, 110 samples collected during this period were whole-genome sequenced by our novel cost-effective tiled-amplicon sequencing approach. The designed protocol was inspired by the ARTIC protocol, which was implemented to sequence millions of SARS-CoV-2 samples worldwide [13]. Novel sequences were compared with publicly available sequences from other geographic locations and from more recently circulating strains in Japan. The results of this multi-year comparison allowed us to assess the sustained evolution of a reduced number of strains limited to Japan against an alternative flow of different variants from geographically neighboring countries.

## 2. Materials and Methods

### 2.1. Sample Collection and Extraction

Clinical samples from a sentinel clinic in Japan were collected between 2011 and 2020 [14]. Viruses were isolated, genomic DNA was extracted, and the NIID provided the material under a Material and Transport Agreement. These samples were collected as part of surveillance for infectious respiratory agents conducted in Japan [6]. The original classification of the infections was performed by partial sequencing following the protocol by Okada et al. [15]. Among the samples initially classified as HAdV-3 with sufficient viral load, 110 were randomly selected for whole-genome sequencing. The genetic material of the viruses was extracted using the High Pure Viral Nucleic Acid Kit [Roche] (Roche, Basel, Switzerland).

### 2.2. Tiled-Amplicon Whole-Genome Sequencing and Base-Calling

The primers for the HAdV-3 tiled-amplicon whole-genome sequencing were designed following the approach used for SARS-CoV-2 and monkeypox virus genome sequencing [16,17]. In short, our implementation used a set of reference sequences (Supplementary Table S1) that were multiple-sequence aligned as input to PrimalScheme (https://primalscheme.com/), with parameters for two primer pools with an amplicon average length of 2500 nucleotides (nt). The conservation of primers across the reference sequences was further assessed to select among potential designs, and an additional 400 nt amplicon was added to cover more of the genome upstream of the E1 region. The sequences of the designed primer sets are shown in Table 1, including their intended non-overlapping pools. The primer pairs were evenly mixed within their assigned pools to a final concentration of 10 μM per pool. The tiled amplicons were amplified using Q5 Hot Start High-Fidelity 2X Master Mix (M0494L, New England BioLabs, Ipswich, MA, USA) as follows: two reactions, corresponding to the two pools, were prepared per sample. Each reaction mixes 12.5 μL 2X Master Mix, 2.5 μL primer pool mix at 10 μM, 2 μL sample DNA, and 8 μL nuclease-free water (NFW). The amount of sample DNA was set for our isolated DNA concentrations, 34.3 ± 4.7 ng/μL (mean ± S.E.) (Supplementary Table S2). Both reactions were set in the thermocycler with the following thermal cycling program: 98 °C for 30 s, followed by 35 cycles of 98 °C for 10 s, 68 °C for 20 s, and 72 °C for 100 s. Then, the program closes with a final extension at 72 °C for 120 s, followed by a hold at 4 °C for 30 s. The protocol takes advantage of multiplexing the sequencing using the Oxford Nanopore Technologies (ONT) Rapid Barcode (SQK-RBK114.96, ONT, Oxford, UK) with batches of eight barcodes sequenced in Flongle flow cells (FLO-FLG114, ONT); hence, pools of amplicons were combined per sample and quantified, subsampled to ca. 50 ng in 10 μL NFW per sample and barcoded with distinguishable barcodes per batch following the manufacturer’s instructions. The barcoded mixtures were combined and resuspended following the protocol for AMPure XP beads to a final amount of 15 μL in elution buffer. The batch of combined barcodes was sequenced in a Flongle flow cell, capturing the data for 24 h per batch. The collected data was base-called using Dorado v0.8.3 choosing the ‘DNA—400 bps—5 kHz’ chemistry and ‘Super-accurate base-calling’; also, the data was demultiplexed by barcoding with ‘SQK-RBK114.96’ as barcode kit and trimming the barcodes from the reads. This protocol uses ONT products for sequencing due to their low cost, short analysis time, and long reads, reducing the number of primers required; however, a similar approach can be used with other sequencing technologies if sequence accuracy is a concern.

### 2.3. Genome Assembly, Quality Control, and Type Classification

The genome assemblies were performed by aligning the reads against reference genomes of HAdV-3 (GenBank Acc. No. AY599834) and HAdV-1 (Acc. No. AC_000017) with minimap2 [18] with parameter ‘map-ont’. The aligned reads were used to analyze the depth and coverage per sample and generate consensus sequences with samtools [19]. Additionally, de novo assembly was performed with SPAdes [20] to search for evidence of potential mixed types in the same isolated samples; thus-assembled contigs were matched to the closest adenovirus genome in a local database using BLAST [21] against the reference genomes for HAdV-3 and HAdV-1.

### 2.4. Phylogenetic and Molecular Clock Analyses

Multiple-sequence alignments of reference sequences (Supplementary Table S1) and sequences generated in this study were performed with MAFFT using the FFT-NS-I algorithm [21]. Phylogenetic trees were inferred by maximum-likelihood (ML) approaches with IQ-TREE version 2.3.1 [22] and the support for the topology tested with SH-like approximate likelihood ratio test (SH-aLRT) and ultra-fast bootstrap (UFBOOT) with 10^4^ replicates and considering support for branches with values >80% and >95%, respectively.

Multiple-sequence alignments of coding sequences for DNA polymerase, pTP, DBP, and hexon were generated considering all HAdV-3 sequences. These alignments were used to infer the molecular clock and migratory patterns using a Bayesian approach with BEAST X v10.5. The inference included an analysis of the phylogeographic origin of the sequences, following an approach similar to that of Lemey et al. [23]. The BEAST chains were set to 5 × 10^7^ states, considering different combinations of models: molecular clocks (strict or uncorrelated relaxed Hamiltonian Monte Carlo) with population models, which were constant, exponential growth, or Hamiltonian Monte Carlo SkyGrid with the number of parameters as 50 and the time at the last transition point set to 10.0. The models were calibrated using the collection years and countries of the samples. Additionally, the tree topologies inferred along the Markov chain of states were used as input to calculate the frequency of jumps between countries and the time of persistence in each country.

### 2.5. Sliding Window Analysis and Nucleotide Composition Analysis

The distribution of divergence among sequences, the percentage of guanine and cytosine (%GC), and the ratio of transitions to transversions (κ = Ts/Tv), were assessed along the aligned genomes in a sliding-window fashion with window and step lengths of 100 nt and 20 nt, respectively. All genome annotations follow the HAdV-3 reference (Acc. No. AY599834).

### 2.6. In Silico Restriction Fragment Length Polymorphism Profiles

To provide context on the potential analysis of the samples under a restriction fragment length polymorphism (RFLP) analysis, the potential digestions of the sequenced genomes were compared and mapped to the genomes using the enzyme recognition patterns of enzymes BamHI (5′-G|GATCC-3′), BglII (5′-A|GATCT-3′), HindIII (5′-A|AGCTT-3′), SmaI (5′-CCC|GGG-3′), and XhoI (5′-C|TCGAG-3′). Sequences were clustered by pattern distance using the Jaccard index.

### 2.7. Ethical Statement

The study was conducted in accordance with the Declaration of Helsinki. The study protocol, including specimen collection and testing, was approved by the Ethics Committee of the National Institute of Infectious Diseases (Approval No. 1482, approved on 20 December 2022) and by the JMA Ethics Review Committee of the Japan Medical Association (No. 30-6, approved on 26 March 2024). Consent for analysis was obtained at the time of specimen collection. Samples were collected at the NIID as part of the surveillance of respiratory pathogens in Japan [24]. All clinical and patient data have been removed before isolation.

## 3. Results

### 3.1. Epidemiological Context of HAdV-3 in Japan

**Figure 1:**
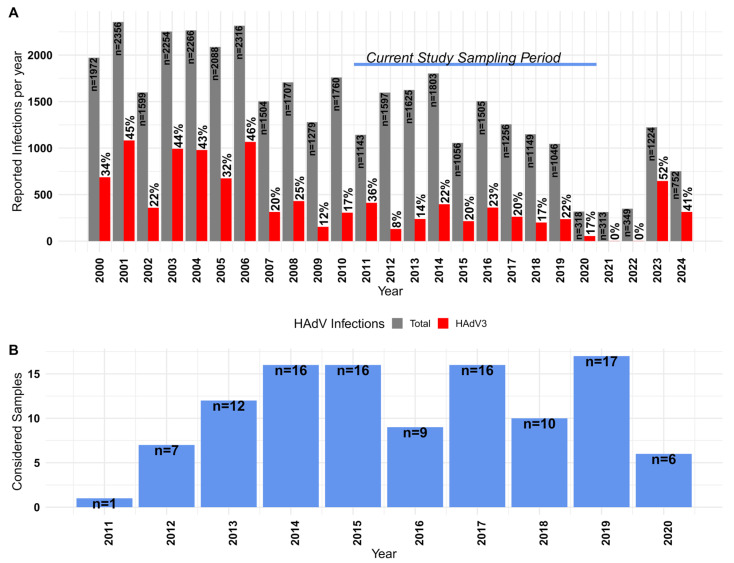
Adenovirus cases reported in Japan between 2000 and 2024. (**A**) The horizontal and vertical axes show the year and number of reported cases per year, respectively. The gray bars show the total number of reported adenovirus cases per year, with the number annotated for each bar. The red bars show the number of cases attributed to HAdV-3, with annotations showing the percentage relative to the year. Data compiled from the IASR reports available at: https://id-info.jihs.go.jp/en/surveillance/iasr/index.html accessed on 10 February 2025. (**B**) The number of samples considered per year for the study (vertical axis) is annotated in each bar representing the year (horizontal axis).

Between 2000 and 2024, HAdV-3 infections in Japan have been reported and tracked alongside other adenovirus infections, with occasional outbreaks. The Infectious Agents Surveillance Report (IASR, available at https://id-info.jihs.go.jp/en/surveillance/iasr/index.html, accessed on 10 February 2025) recorded history was assessed for the frequency and burden of adenoviral cases due to HAdV-3 in Japan for the last 25 years (Figure 1A). Furthermore, the clinically reported yearly average number of adenovirus infections was 1449.0 ± 606.4 (95% CI: 1199.2–1699.8), among them HAdV-3 represented on average the 26 ± 14% (95% CI: 20.0–32%) with 421.0 ± 326 cases yearly. Considering this percentage of yearly reported cases attributed to a single adenovirus type and the infectious burden to public health, we focused on 110 samples collected and isolated between 2011 and 2020 (Figure 1B).

### 3.2. HAdV-3 Tiled Amplicon Approach for Whole-Genome Sequencing

**Figure 2:**
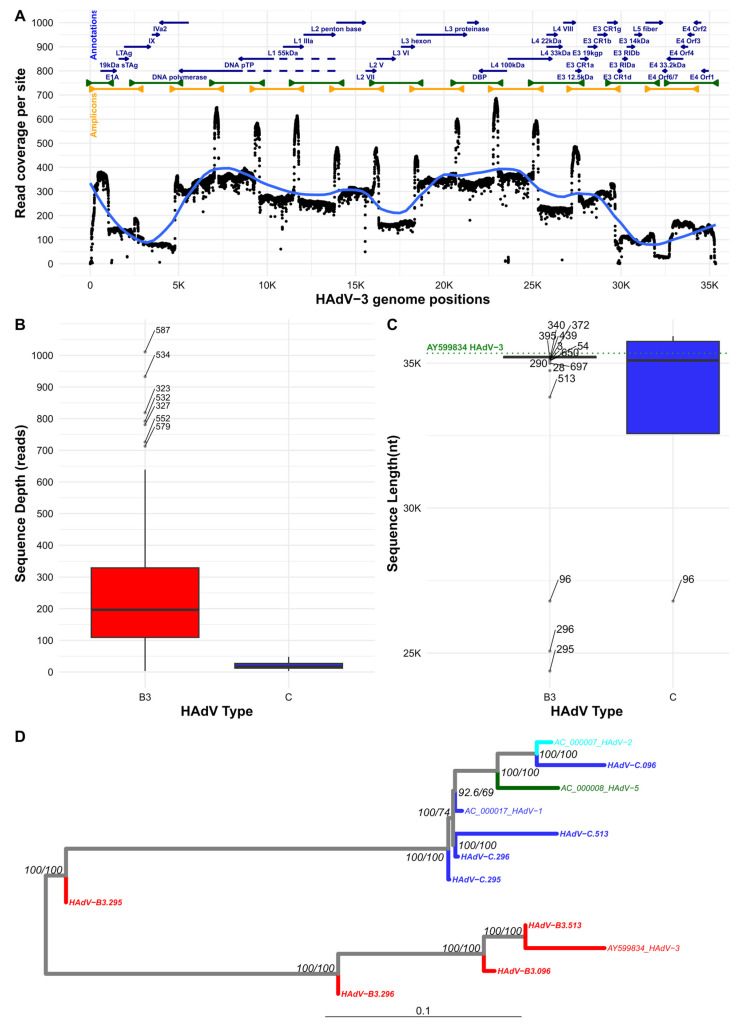
HAdV-3 sequencing results. (**A**) The average sequencing read coverage per position (vertical axis) across the HAdV-3 genome (horizontal axis) for all samples is represented by the black dots. The average coverage per 1000 sites is shown in blue. On top of the panel, the distribution of amplicon tiles for pools 1 (green) and 2 (yellow) is shown, with the protein product annotation corresponding to the positions in the HAdV-3 reference (AY599834) below. (**B**) Distribution of average sequence depth coverage in reads grouped by types. (**C**) Distribution of achieved lengths for sequenced samples in types 3, 1, and 2. The length of the complete HAdV-3 genome has been added for reference as the dotted green line. The labels of the outliers are shown for (**B**,**C**). (**D**) ML phylogenetic tree of sequences from samples showing co-infections for both species. Assembled sequences are shown and colored according to the genome reference sequences for *Mastadenovirus caesari* (HAdV-C) types 1 (blue), 2 (light blue), 5 (green), and HAdV-3 (red). Branch nodes are annotated with the SH-aLRT/UFBOOT support as percentages.

We designed primers across the HAdV-3 genome following a tiled amplicon approach with overlapping amplicons averaging sizes of 2500 nt (Table 1) (Figure 2A). Despite differences in the average coverage across the nucleotide sites of samples, the overall read depth for the HAdV-3 sequences was 262.6 ± 192 reads (Figure 2B). On the other hand, most of the sequences had genome lengths excluding undefined nucleotides, i.e., N’s, nearing the length of the reference genome, i.e., 35,345 nt, (Acc. No. AY599834) with average length 34,802 ± 1861 nt or ca. 98.5% of the expected length (Figure 2C). Nevertheless, the genome sequences encompassed all the encoding genes for the canonical adenovirus proteins, as the regions with lower depth were located towards the terminal regions of the genomes. The approach provided overall good sequence depth (Figure 2B) and genome coverage (Figure 2C) for 105 out of 110 samples considered in this study.

Among the five samples with poorer sequencing performance (Supplementary Table S2), de novo assembly revealed evidence of coinfection with members of other adenovirus species, *Mastadenovirus caesari* (HAdV-C), in four samples. Conservation of primers across both species enabled partial sequencing of the additional pathogens identified as type 1 (Figure 2B,C). Such results highlighted the risk of underreporting the circulation of certain types, as current methods rely on partial sequencing of short targets. Phylogenetic analysis of these partial sequences against HAdV-C type references confirmed the identity of the coinfecting strains (Figure 2D), while HAdV-3 sequences from the same samples were also assembled. In detail, the identity of the assembled sequences to HAdV-3 was 90.5 ± 6.0%, while the identity to HAdV-C type 1 of assembled HAdV-C sequences was 97.1 ± 2.25%. The fifth sequence with poorer performance was attributed to the low sample concentration of 5.8 ng/μL.

### 3.3. Inferred Bayesian Phylogenetic Relation of HAdV-3 Sequences

**Figure 3:**
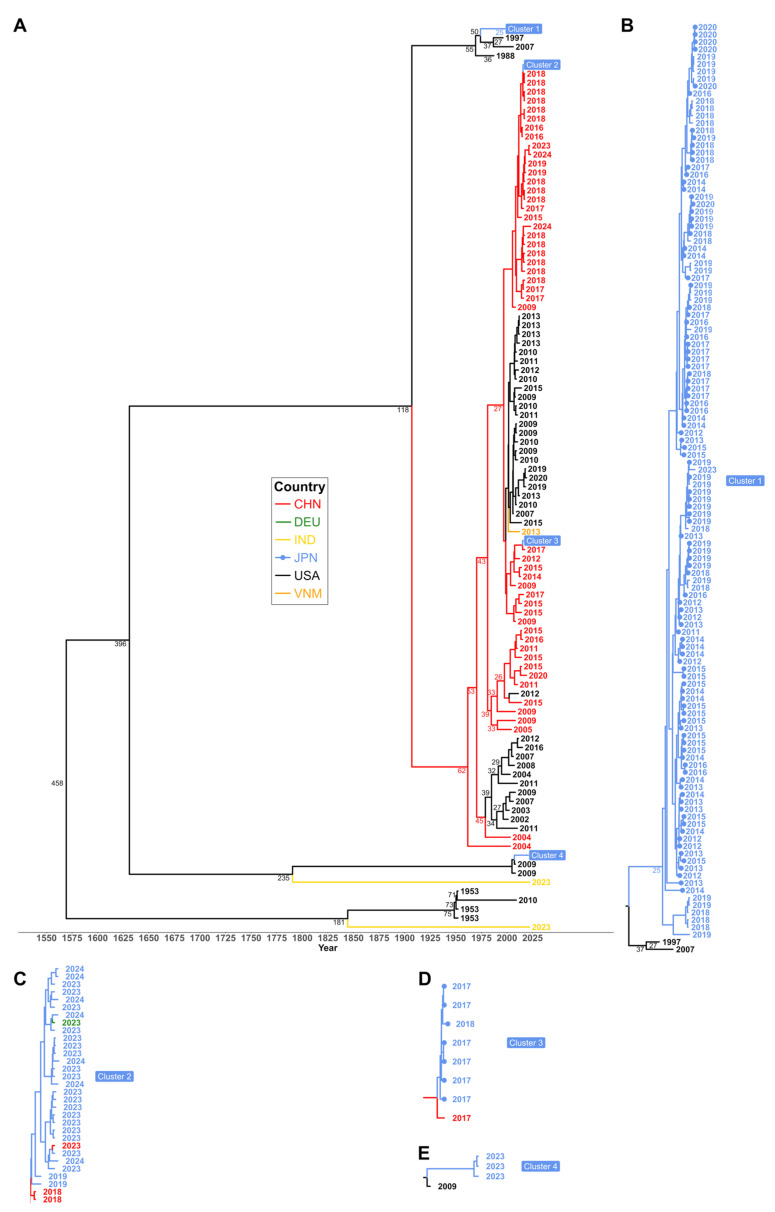
Bayesian phylogenetic tree of HAdV-3. (**A**) The tree was inferred from genomic loci encoding the proteins DNA polymerase, pTP, DBP, and hexon. The calibration used the years of collection as shown in the tip labels. Branching years next to nodes are displayed for divergences older than 25 years. Tips and branches are colored according to the country of collection and the inferred origin, respectively, as shown in the legend. Clusters of Japanese sequences are collapsed into clusters 1–4, as shown in detail in panels (**B**–**E**), respectively. Samples sequenced in this project are marked with a disk at the tip.

The assembled HAdV-3 genomes were compared against other publicly available sequences of the same adenovirus type with Bayesian approaches to infer the origins of the phylogenetic variants circulating in Japan during the analyzed period (Figure 3). In consideration of effects of potential unaccounted recombination events, the molecular clock was based on the genes of the replication machinery and the major serological determinant shared by all sequences in the same type, i.e., the hexon. The phylogenetic inference was performed considering the genes encoding the DNA polymerase, terminal protein (pTP), DNA-binding protein (DBP), and the hexon protein. The mutation rate was estimated at 1.75 × 10^−5^ ± 2.4 × 10^−6^ mutations/site/year, within the range expected for a double-stranded DNA virus [25]. The time to the most recent common ancestor (tMRCA) of all considered samples was estimated at 458 years ago, with a 95% high posterior density (95% HPD) in the range [342, 575] years. However, 96% of analyzed sequences (*n* = 249/260), including the new sequences, diverged in the last 118 years.

The samples sequenced in this project were segregated into two of the four distinguishable clusters of Japanese sequences (Figure 3B–E). Both clusters contained genomes sequenced independently by other teams, confirming our assembled genomes matched strains circulating in Japan. Nevertheless, a significant difference in the sampling years of the Japanese sequences was observed between clusters 2, 3, and 4. In detail, cluster 1 contained more samples collected before 2020 (117 out of 124), while clusters 2 and 3 contained more samples collected after 2022 (25 out of 34 Japanese sequences; *p* < 1.4 × 10^−15^, Fisher’s exact test). Although cluster 3 contained a small group of sequences from 2017 to 2018 (*n* = 7), the reason for the significant difference in the composition of collection times between the clusters remains unclear. The tMRCA for both clusters was estimated to be 118 years ago, with a 95% HPD interval of [83, 156] years. On the other hand, cluster 4 corresponds to a set of sequences from 2023 (*n* = 3) clustering with a branch of HAdV-3 that diverged from the branches leading to the other two clusters approximately 395 years ago (95% HPD [290, 507] years). The contrast with sequences from different countries suggested that phylogenetic clusters 2 and 3 have circulated globally for the last 25 years. Remarkably, the tMRCA of Japanese sequences in cluster 2 was approximately 27 years (Figure 3A), and their clustering with contemporaneous strains collected in China indicated the flow of strains across the Asian region.

We performed an in silico RLFP analysis to identify potential patterns for economic tracking of these variants (Supplementary Figure S1). The profiles largely followed the phylogenetic analysis, showing two major variants (Supplementary Figure S2). The comparison provided support for at least two enzymes, BglII and SmaI, with cleavage patterns distinguishing the two variants.

**Figure 4:**
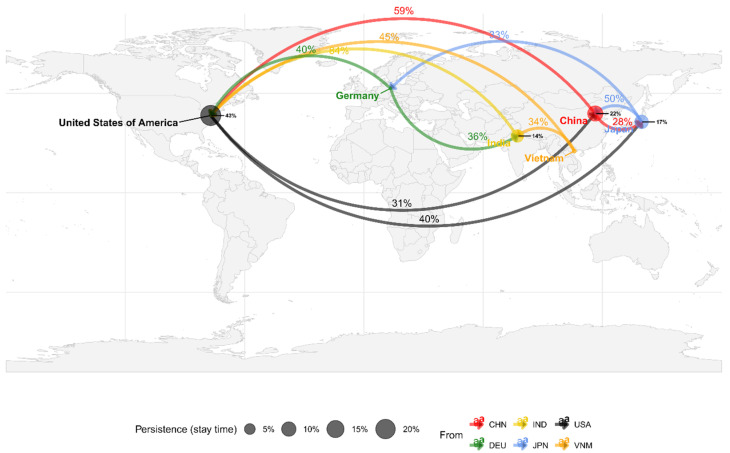
Inferred migration of HAdV-3 across countries with genome sequences. The map shows the countries with publicly available sequences. The frequencies of virus migratory jump events between countries (arrows) and the percentage of time persisting in the country (disks) were inferred from the Bayesian analysis. The frequencies represent the number of inferred trees reflecting the migration of the variants. The jumps with support greater than 25% and persistence time higher than 10% of the total length of the tree are shown next to the corresponding events. The jumps and disks are colored according to the legend at the bottom. Disks are sized according to the persistence time of strains in each country.

The Bayesian phylogenetic inference was further analyzed to summarize the frequency of the inferred “jumps” of strains between the different countries and the percentage of time that variants persisted in different countries afterwards (Figure 4). The generated summary of Bayesian trees, a product of the inference, substantiated the perception of a strong establishment of the strains in the different countries; in contrast, frequent transmission among countries would have been expected to be shown as more diversified clades in the phylogenetic tree (Figure 3A) and more edges with lower support in the inferred geographical migration (Figure 4). Two major introduction events to Japan were inferred from the Bayesian analysis related to variants circulating in China and the United States of America. It is also noted that, after introduction, these variants have been repeatedly detected and have diverged into the clusters mentioned above.

### 3.4. Uneven %GC Content Distribution

**Figure 5:**
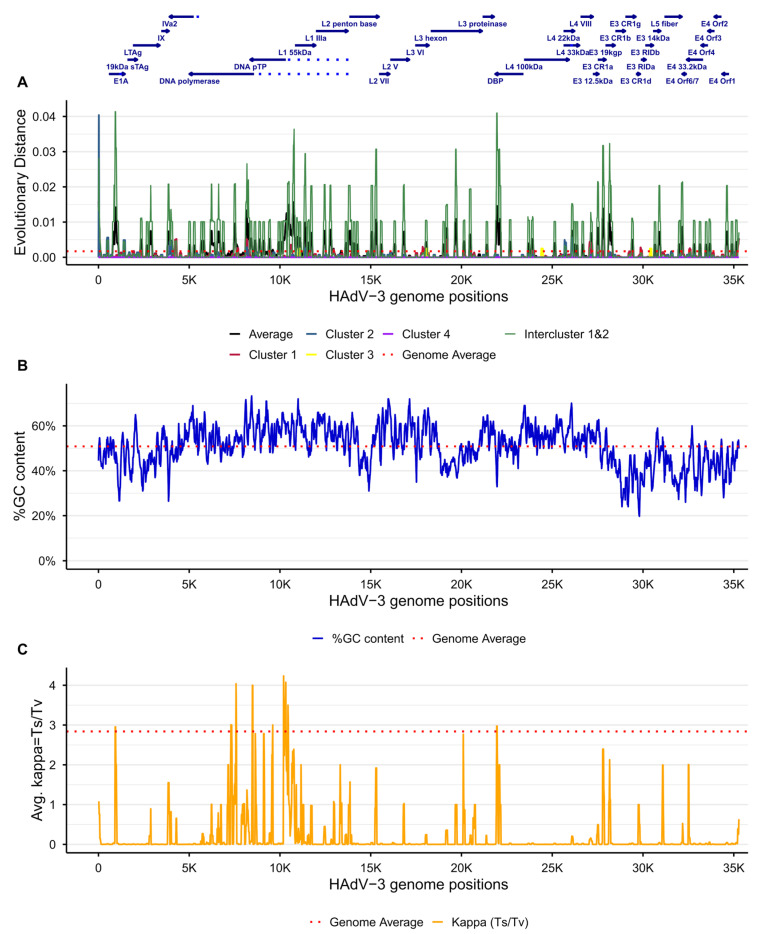
Sliding window analysis of the divergence distribution across the HAdV-3 genomes. The horizontal axes are aligned with the annotations in the top panel and show positions along the genome. (**A**) The vertical axis shows the average evolutionary distance per window across all sequences in clusters 1 and 2 of Figure 3, and between both, as indicated in the legend at the bottom of the panel. (**B**) The vertical axis shows the %GC per window. (**C**) The vertical axis shows the average ratio of transitions to transversions (κ = Ts/Tv) per window. In all panels, the average observed per genome is shown as dotted lines.

The Bayesian phylogenetic inference among the considered sequences also estimated κ = 5.43 ± 0.54, the transition-to-transversion ratio (A↔G, C↔T) to (A↔C, A↔T, G↔C, G↔T). This value was considered moderately high and could indicate a mutational bias. To test such a hypothesis, the distribution of mutations, percentage of guanine/cytosine nucleotides (%GC) and κ along the genome were explored with sliding window analyses (Figure 5). The evolutionary distance analysis along the genome showed a distribution with discrete peaks of mutations along the genome (Figure 5A). Nevertheless, the distance analysis by clusters and inter-cluster between sequences in cluster 1 and 2 (Figure 3B,C) suggested most of the average evolutionary distance among sequences was due to inter-cluster divergence.

On the other hand, the %GC distribution suggested an uneven distribution of the %GC content, despite the genome %GC being ~50.8% (Figure 5B). Furthermore, areas of lower %GC overlapped with areas encoding genes related to host-interactions, such as the epitope determinants in the penton base, hexon, and fiber. Also, relatively lower %GC in genes involved in immune evasion in the E3 transcriptional region, and replication modulation in E1 and E4 transcriptional regions. Genomic regions with higher %GC content matched loci with higher κ ratios (Figure 5C), suggesting that the high value observed in the Bayesian inference could be due to the selection of genes in the molecular clock model, i.e., DNA polymerase and pTP. Therefore, we repeated the Bayesian inference considering the complete genomes to rule out the possibility that such composition bias could affect the results. Although the second Bayesian inference yielded a lower κ value, it remained moderately high at κ = 4.96 ± 0.25. Therefore, a potential mutational bias was suggested for the genome.

## 4. Discussion

This report presents a novel, cost-effective HAdV-3 sequencing protocol that enables near-whole-genome sequencing of multiplexed samples in a short time with ONT devices. The genome coverage and sequence depth generate sufficient raw data to assess and validate sequence quality and further analyze variation across the genome. The time required to prepare samples for sequence analysis after DNA extraction can be as short as 1 day, enabling quick turnaround even in diagnostic settings. This accessible approach allows answering epidemiological queries about the prevalence, transmission, and endemicity of HAdV-3 in Japan. We further used this method to examine the relationship among samples collected in Japan (*n* = 105) in the context of yearly outbreaks.

Our findings supported the hypothesis of the endemic circulation of phylogenetically closely related HAdV-3 variants in Japan between 2011 and 2020, as evidenced by the majority of samples clustering together for over a decade. Nevertheless, comparisons with samples sequenced after 2023 suggested a change in the predominant variant following the COVID-19 pandemic, as reported for other respiratory viruses [26] and even other adenovirus species [27]. This change in variant predominance could be attributed to a decrease in HAdV-3 transmission, an indirect effect of the non-pharmaceutical interventions introduced to reduce COVID-19 incidence. Consequently, after the interventions were rolled back, different HAdV-3 variants still circulating in Japan occupied the epidemiological niche vacated by the previous predominant strain in a founder effect. This interpretation was further supported by the coexistence of sequences from clusters 1 and 3 during 2017–2019, with members of clusters 2 and 4 not increasing in frequency until after the COVID-19 pandemic. Although these results were consistent with sustained HAdV-3 transmission in Japan with yearly outbreaks, they also raised the hypothesis of a potential uncharacterized epidemiological process of maintained subclinical transmission.

Developing appropriate tools for epidemiological studies, such as our sequencing protocol, provides impactful data for assessing the transmission and prevalence of different pathogens. These data can be used to reconstruct the context of distinct outbreaks. Also, it can serve as input for risk assessment and timely planning interventions to mitigate the impact of pathogens on public health. Currently, only two licensed adenovirus vaccines are available to protect against types 4 and 7, with usage limited to USA military personnel [28]. Therefore, generating more detailed epidemiological and genomic data could provide a basis for developing novel vaccines against additional adenovirus types for the general population.

The long-read sequencing in our protocol enabled the detection of different types in the same sample. Moreover, partial sequencing of HAdV-C strains in respiratory infection samples indicates the prevalent co-circulation of the members of both species in Japan and the risk of underreporting co-infections, as reported in other Asian countries [29]. Although the amplicon primers were specifically designed for members of the HAdV-B species, the relatively high interspecies conservation across loci allowed sufficient recovery of viral genome to identify a strain in HAdV-C. Also, alternative primer versions can be added to the pools for different target species. Nevertheless, this report provides proof of concept for the simultaneous genomic characterization of multiple adenovirus types in a single sample. Consequently, these new protocols could facilitate more detailed clinical profiling of co-infections and their effects on the severity and progression of patient infections. Accurate HAdV-3 sequencing would also enable exploration of coinfections with other respiratory viruses, such as influenza and respiratory syncytial viruses [3].

The uneven distribution of nucleotide composition and its relation to evolutionary trends in members of the adenovirus species *Mastadenovirus dominans* (HAdV-D) has been previously characterized [10]. Although the analysis of HAdV-3 sequences suggested a moderately high ratio of transitions to transversions, the distribution of such mutations seemed more localized in loci with relatively high %GC. In contrast, loci encoding proteins that directly interact with the host showed a relatively low %GC compared with the rest of the genome. This observation suggests distinct selective pressures arising from structural and functional constraints, as previously reported for adenovirus types in HAdV-D [30].

This study was limited by the isolated samples, which represented a random subset of HAdV-3 cases during the sampling period. Further studies could exploit the protocol to widen the number of targeted samples and generate a more complete picture of viral dynamics. Additionally, the availability of samples was related to the severity and classification of the original diagnosis, precluding analysis of subclinical or asymptomatic infections. Other publicly available sequences were included for comparison to ameliorate biases introduced by the serendipity of the chosen samples. Data on the severity of clinical signs or differences between strains was unavailable, hindering deeper analysis on the impact of mutations. On the other hand, although the latest chemistry version of Oxford Nanopore Technologies sequencers is reported to provide high sequence accuracy (>99%) [31], high coverage (>30×) across the genome was pursued to avoid sequence artifacts.

## 5. Conclusions

We report a novel protocol to enable broader epidemiological analysis of the evolution and transmission of HAdV-3. The approach provided insights into the endemic transmission of this virus in Japan over the years and into the changes in prevalence following the COVID-19 pandemic. The availability of this economic sequencing technique is envisioned to aid in the development of more accurate diagnostic tools and to generate sufficient data to develop novel prophylactic alternatives.

## Figures and Tables

**Table 1:** Tiled Amplicon Primers.

No.	Name	Tile	Start ^a^	End ^a^	Length ^b^	Pool ^c^	Sense	Sequence
1	B3_2500_1_LEFT	1	221	243		1	+	TTTTCCCACGCTTACTGACAGG
2	B3_2500_1_RIGHT	1	2699	2722	2501	1	−	CCCCAAGCTTCTACACACGTATT
3	B3_2500_3_LEFT	3	4778	4800		1	+	GGGACCGTAAATGACCCCAATT
4	B3_2500_3_RIGHT	3	7248	7270	2492	1	−	GTTACTTTCGCTTTGCCCAACC
5	B3_2500_5_LEFT	5	9302	9324		1	+	ATCTTTCAATGACCTCTCCGCG
6	B3_2500_5_RIGHT	5	11,774	11,796	2494	1	−	GGTGCATCCTGTCATTGCGATA
7	B3_2500_7_LEFT	7	13,796	13,817		1	+	AGATTCGGGCGCATGTTGTAA
8	B3_2500_7_RIGHT	7	16,294	16,317	2521	1	−	CAGCCCATCATCGTCATCTTCTT
9	B3_2500_9_LEFT	9	18,321	18,343		1	+	GCGCTTAACTTGCTTGTCTGTG
10	B3_2500_9_RIGHT	9	20,826	20,848	2527	1	−	GTGACGGCTTTGTAGTCAGTGT
11	B3_2500_11_LEFT	11	22,764	22,786		1	+	GCCTTCATAATCGGTGCATCCA
12	B3_2500_11_RIGHT	11	25,308	25,330	2566	1	−	AGTGGTAGGAGAGGTAGTTGGC
13	B3_2500_13_LEFT	13	27,198	27,220		1	+	TCTTCCTTCACTCCTCGTCAGG
14	B3_2500_13_RIGHT	13	29,654	29,676	2478	1	−	ACTACCACGGCAGTAATGATGC
15	B3_2500_15_LEFT	15	31,626	31,650		1	+	CCCCCTAACAAAGTCAAACCATTC
16	B3_2500_15_RIGHT	15	34,090	34,112	2486	1	−	CCGGGACCTGTTTGTAATGTGT
17	B3_1000_1_LEFT	1	59	78		2	+	AAAAAGTGCGCGCTGTGTG
18	B3_1000_1_RIGHT	1	1025	1047	988	2	−	GCTCCGGACAGTCCAACTTAAA
19	B3_2500_2_LEFT	2	2490	2512		2	+	ACATATCAGGGAATGGGGCAGA
20	B3_2500_2_RIGHT	2	4942	4964	2474	2	−	AAAAACTTCTCCTCGCTCCAGG
21	B3_2500_4_LEFT	4	7011	7033		2	+	GAAACCCGTCTTTTTCTGCACG
22	B3_2500_4_RIGHT	4	9559	9581	2570	2	−	AAACAGTGCTCAGCCTACCTTG
23	B3_2500_6_LEFT	6	11,515	11,537		2	+	GTTGAACATCACCGAGCCTGAT
24	B3_2500_6_RIGHT	6	14,050	14,072	2557	2	−	ACGAATGCTGTTTCTCCCTTCC
25	B3_2500_8_LEFT	8	16,052	16,074		2	+	AAGAGGCAATGTGTACTGGGTG
26	B3_2500_8_RIGHT	8	18,515	18,537	2485	2	−	TGAAGTAGGTGTCTGTTGCACG
27	B3_2500_10_LEFT	10	20,647	20,669		2	+	GGAAGGATACAACGTGGCACAA
28	B3_2500_10_RIGHT	10	23,031	23,053	2406	2	−	CATGGAAGTGATGGCTGTGCTA
29	B3_2500_12_LEFT	12	25,024	25,046		2	+	AGCTCTTGCAGAGATCCCTCAA
30	B3_2500_12_RIGHT	12	27,621	27,643	2619	2	−	ATGGTTGTATTTCCCTGGTCGC
31	B3_2500_14_LEFT	14	29,393	29,415		2	+	GCAACGGAAGAGACTTGACCAT
32	B3_2500_14_RIGHT	14	31,880	31,902	2509	2	−	TCTGAGGCTCCCATTAGCGTTA
33	B3_2500_16_LEFT	16	32,715	32,737		2	+	TGCGACTGCTGTTTATGGGATC
34	B3_2500_16_RIGHT	16	35,207	35,228	2513	2	−	GACCGTGGGAAAATGACGTTG

^a^ Positions relative to the reference genome acc. no. AY599834. ^b^ Tile length calculated between left and right primers for the tile relative to the reference genome. ^c^ Set of primers to be combined in the same pool mixture.

## Data Availability

New genome sequences were deposited in DDBJ (LC879946-53, LC882547-652).

## Supplementary Materials

The following supporting information can be downloaded at: https://www.mdpi.com/article/10.3390/v18010074/s1, Figure S1: In silico restriction fragment length polymorphism (RFLP) profiles; Figure S2: Maximum-likelihood phylogenetic tree of HAdV-3 annotated with the RFL profiles from A to H; Figure S3: HAdV-3 genome mapping digestions under BglII and SmaI enzymes; Table S1: Metadata of reference sequences; Table S2: Sequenced samples details.

## Abbreviations

The following abbreviations are used in this manuscript:

HAdV-3	Human adenovirus type 3
HAdV-B	*Mastadenovirus blackbeardi*
HAdV-C	*Mastadenovirus caesari*
NIID	National Institute of Infectious Diseases
pTP	Pre-terminal protein
DBP	DNA-binding protein
RFLP	Restriction fragment length polymorphisms
ONT	Oxford Nanopore Technologies
ML	Maximum-likelihood
IASR	Infectious Agents Surveillance Report
HPD	High-posterior distribution
tMRCA	Time to the most recent common ancestor
CHN	China
DEU	Germany
IND	India
JPN	Japan
USA	United States of America
VNM	Vietnam
