# Supplementary material for: Tiled-Amplicon Whole-Genome Sequencing Method Reveals Endemic Circulation of Human Adenovirus Type 3 in Japan
Source: Viruses. 2026 Jan 5;18(1):74. doi: 10.3390/v18010074 (PMC12846424; doi:10.3390/v18010074)
Supplement: Supplementary file 1 [file viruses-18-00074-s001.zip › Supplementary Materials.pdf]

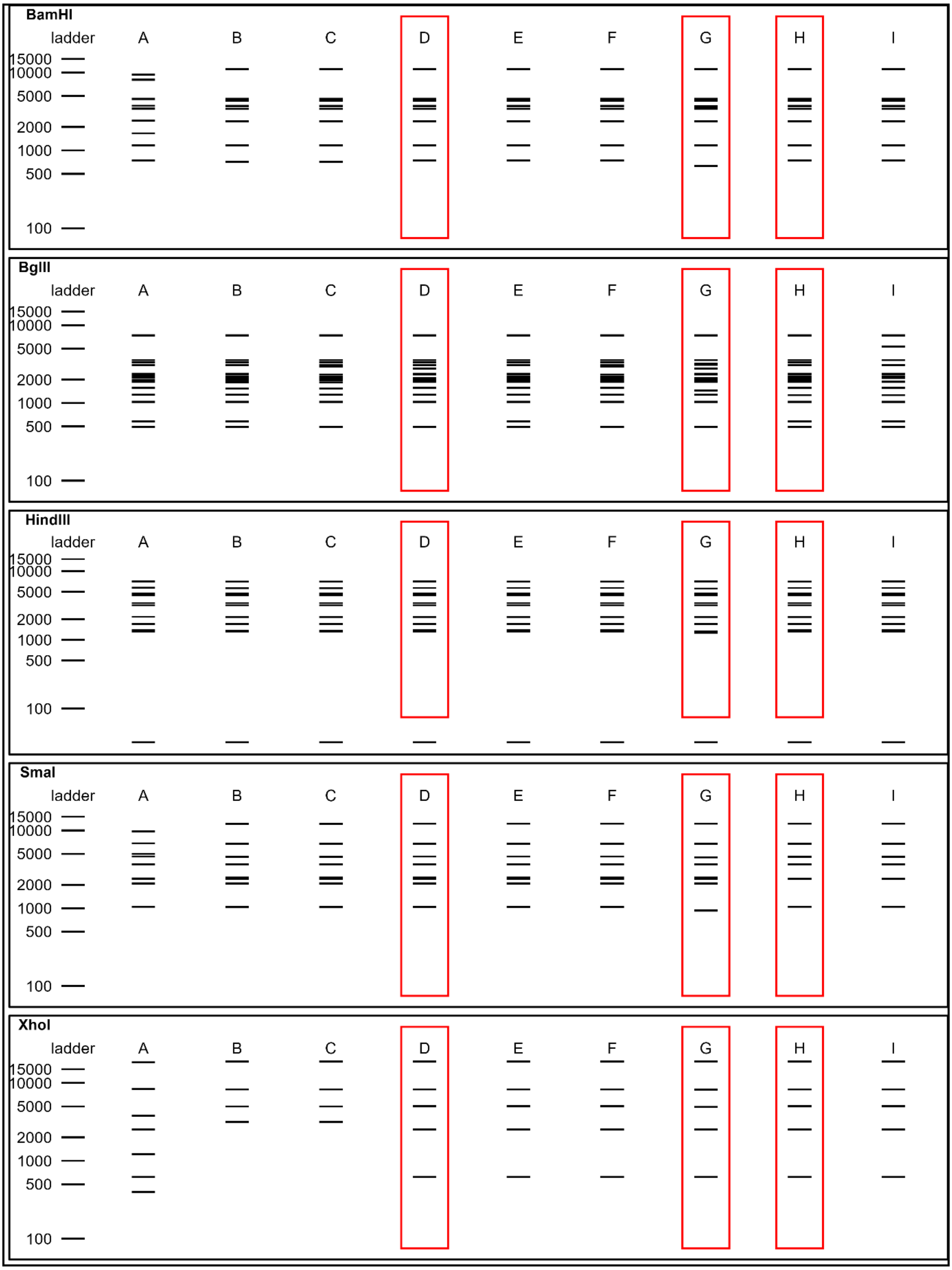

Supplementary Figure S1. In silico restriction fragment length polymorphism (RFLP) profiles. Each panel shows the expected digestion pattern according to the enzyme on the top left. The vertical axis is shown as logarithmic values similar to the agarose migration of bands according to the fragment length. The profiles D, G, and H, corresponding to sequences reported in this study, are highlighted in red (see Supplementary Figure S2).

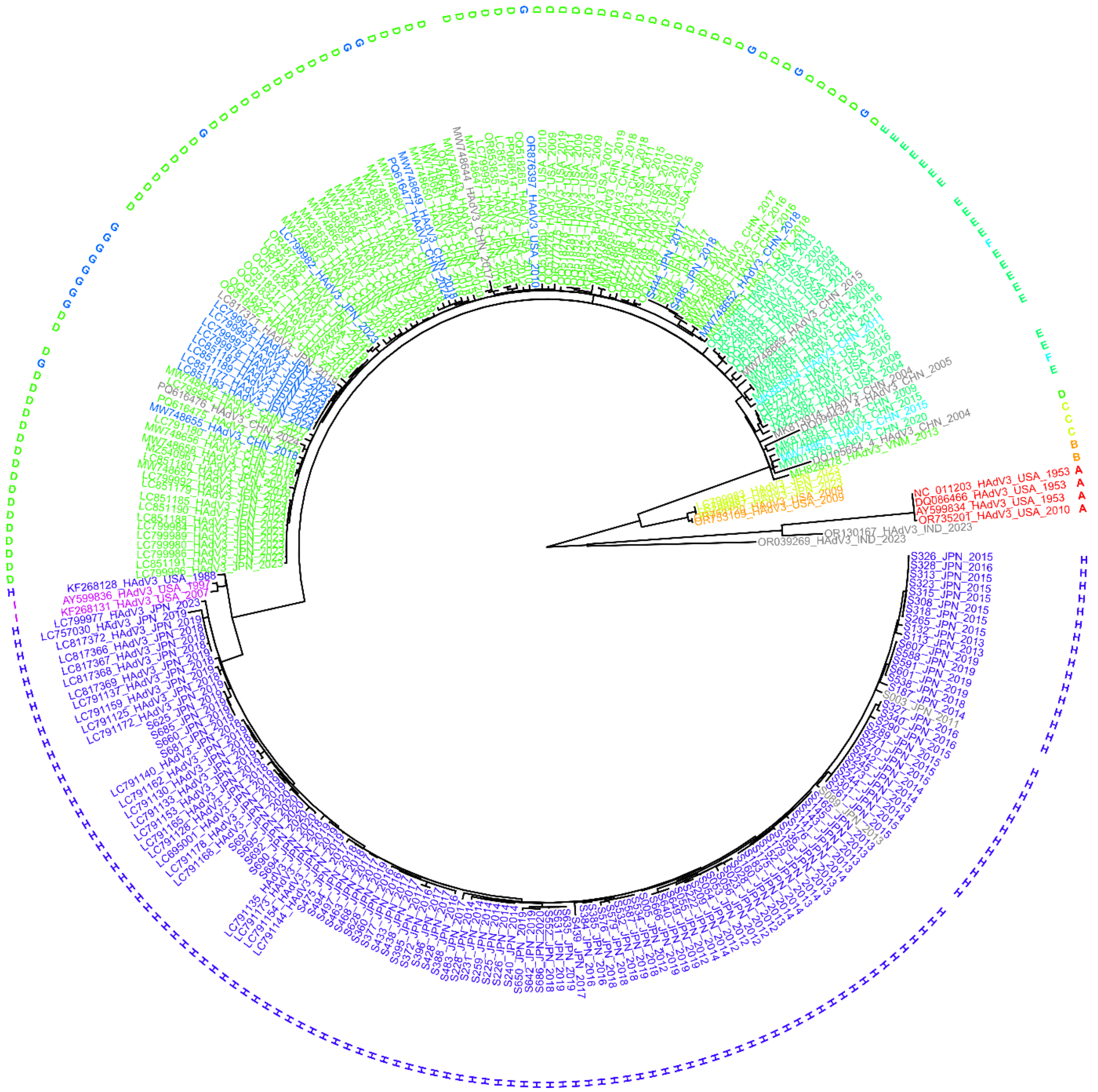

Supplementary Figure S2. Maximum-likelihood phylogenetic tree of HAdV-3 annotated with the RFLP profiles from A to H. Annotations correspond to profiles shown in Supplementary Figure S1. RFLP profiles with a single sequence were omitted from the analysis for readability.

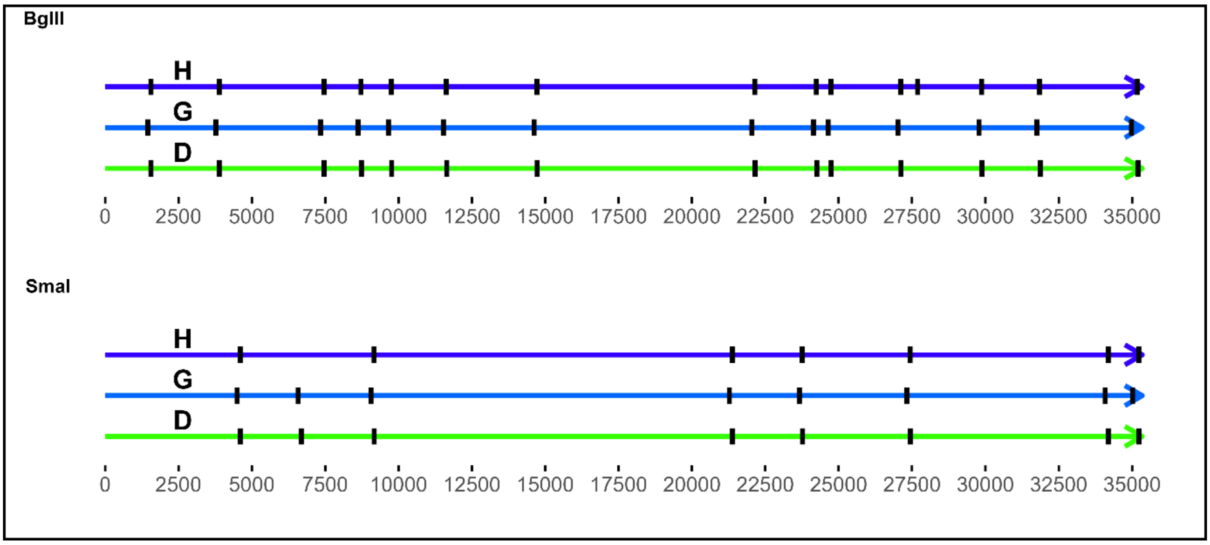

Supplementary Figure S3. HAdV-3 genome mapping digestions under BglII and SmaI enzymes. The horizontal axis represents the genome positions. Each arrow represents one genome sequence per RFLP profile, with the approximate locations of the digestion sites under the corresponding enzyme.
